# The Interface of Tumour-Associated Macrophages with Dying Cancer Cells in Immuno-Oncology

**DOI:** 10.3390/cells11233890

**Published:** 2022-12-02

**Authors:** Isaure Vanmeerbeek, Jannes Govaerts, Raquel S. Laureano, Jenny Sprooten, Stefan Naulaerts, Daniel M. Borras, Damya Laoui, Massimiliano Mazzone, Jo A. Van Ginderachter, Abhishek D. Garg

**Affiliations:** 1 Cell Stress & Immunity (CSI) Lab, Department of Cellular and Molecular Medicine, KU Leuven, 3000 Leuven, Belgium; 2 Laboratory of Dendritic Cell Biology and Cancer Immunotherapy, VIB Center for Inflammation Research, 1050 Brussels, Belgium; 3 Laboratory of Cellular and Molecular Immunology, Vrije Universiteit Brussel, 1050 Brussels, Belgium; 4 Laboratory of Tumour Inflammation and Angiogenesis, VIB Center for Cancer Biology, 3000 Leuven, Belgium; 5 Laboratory of Tumour Inflammation and Angiogenesis, Department of Oncology, KU Leuven, 3000 Leuven, Belgium; 6 Laboratory of Myeloid Cell Immunology, VIB Center for Inflammation Research, 1050 Brussels, Belgium

**Keywords:** macrophages, cancer therapy, tumour microenvironment, radiotherapy, chemotherapy, immunotherapy, immunogenic cell death, apoptosis macrophage targeting, TAM heterogeneity

## Abstract

Tumour-associated macrophages (TAMs) are essential players in the tumour microenvironment (TME) and modulate various pro-tumorigenic functions such as immunosuppression, angiogenesis, cancer cell proliferation, invasion and metastasis, along with resistance to anti-cancer therapies. TAMs also mediate important anti-tumour functions and can clear dying cancer cells via efferocytosis. Thus, not surprisingly, TAMs exhibit heterogeneous activities and functional plasticity depending on the type and context of cancer cell death that they are faced with. This ultimately governs both the pro-tumorigenic and anti-tumorigenic activity of TAMs, making the interface between TAMs and dying cancer cells very important for modulating cancer growth and the efficacy of chemo-radiotherapy or immunotherapy. In this review, we discuss the interface of TAMs with cancer cell death from the perspectives of cell death pathways, TME-driven variations, TAM heterogeneity and cell-death-inducing anti-cancer therapies. We believe that a better understanding of how dying cancer cells influence TAMs can lead to improved combinatorial anti-cancer therapies, especially in combination with TAM-targeting immunotherapies.

## 1. Introduction

Tumour-associated macrophages (TAMs) are one of the most dominant tumour resident immune cells. Thus, not surprisingly, they majorly modulate the tumour microenvironment (TME) in many cancer types [1,2]. In most contexts, TAMs are associated with poor patient prognosis primarily due to their proficient immunosuppressive and wound-healing-like activity within the tumours. This promotes a number of pro-tumorigenic processes such as dysregulated angiogenesis, re-modelling of the extracellular matrix, secretion of pro-tumorigenic cytokines or growth factors and facilitation of metastasis [3]. However, depending on the tumoural or therapeutic context, TAMs are also capable of exerting anti-tumorigenic pressures, either autonomously or by fostering the effector/cytotoxic activity of CD8^+^T cells [3]. This contextual behaviour of TAMs, together with their ontologic heterogeneity, underlies the overall complexity of TAMs’ functional impact. Interestingly, TAM heterogeneity exists not only at the intra-tumoural and inter-patient level but also at the cross-species level [4,5]. For instance, whereas experimental evidence subscribes a pro-tumorigenic role for TAMs across almost all rodent tumour models, human tumours have proven to be more complicated, e.g., TAMs can have a positive or mixed (rather than only negative) prognostic impact in patients with high burden tumours such as colorectal cancer (CRC), head and neck cancer (HNC), lung cancer, prostate cancer and oesophageal cancer [6]. Such heterogenous behaviour may originate from the plasticity and adaptability of TAMs in the backdrop of high genetic diversity in human populations.

One of the most dominant activities of TAMs (especially those ‘derived’ from tissue-resident macrophages) entails the active clearance of dying cancer cells (i.e., efferocytosis) that are constitutively formed during aggressive tumour growth [3]. Since such efferocytosis happens in an immunosuppressive TME context, this largely leads to anti-inflammatory TAM activity that has pro-tumorigenic consequences [7]. However, cancer cell death achieved in the context of immunogenic chemo-radiotherapy or active immunotherapy may repolarise the TAMs toward anti-tumorigenic activity [1]. Thus, the interplay between dying cancer cells and TAMs has major implications for immuno-oncology. In the present review, we discuss the pivotal role of the interface between macrophages and dying cancer cells in regulating tumour responses to anti-cancer therapy. We also discuss the significance of this interface for clinical cancer immunotherapy and the translation of macrophage-targeting immunotherapies.

## 2. Tumour-Associated Macrophages: A Bird’s Eye-View

For a long time, TAMs were believed to originate from bone-marrow-derived monocytic precursors, which is, for a lot of murine models, indeed the case [7]. However, tissue-resident macrophages originate from embryonic precursors in homeostatic conditions and sustain, in addition to the bone-marrow-derived precursors, the overall TAMs abundance [1]. When macrophages are recruited to the tumour or derived from the resident subsets within the tissue-of-origin, their phenotype is ‘skewed’ for tumour-specific activity, giving rise to various TAM subsets [8]. TAMs are the most abundant tumour infiltrating cell type and are widely studied due to their role in tumour progression, metastasis and therapy resistance [9]. In the following sub-sections, we provide an introduction to the basic aspects of TAMs, with particular attention to ontological definitions or terminologies typically used in oncology. 

### 2.1. TAM Diversity and Classifications

**Figure 1:**
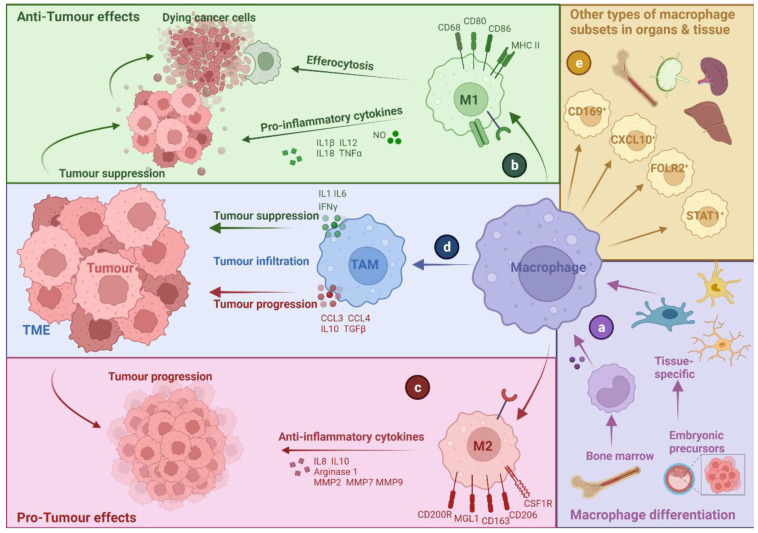
Schematic overview of macrophage development into different classes. Macrophages differentiated from bone marrow and embryonic precursors, different phenotypes will arise according to the tissue origin or organs (**a**). The two main classes of macrophages are classically activated macrophages (M1) and alternatively activated macrophages (M2). Macrophages differentiated into M1-like macrophages express high levels of main histocompatibility complex II (MHC-II) and clusters of differentiation (CD). Once activated, M1-like macrophages can generate nitric oxide (NO) and secrete interleukins (ILs) and tumour necrosis factor α (TNF α). M1-like macrophages will induce an anti-tumour effect and will interact with dying cancer cells by efferocytosis to recruit more immune cells (**b**). On the contrary, M2-like macrophages express multiple CDs, CSF1-receptor (CSF1R) and macrophage galactose-type lecin-1 (MGL1). Once M2-like macrophages are activated, they can secrete IL8 and IL10, matrixmetalloproteinase-2, -7 and -9 (MMP2, MMP7 and MMP9) and arginase, hereby stimulating tumour progression (**c**). Migration and infiltration of macrophages in a tumour environment give rise to various tumour-associated macrophages (TAMs) subsets. TAMs resemble mostly M2-like macrophages by secreting IL-10, TGFβ and C-C motif chemokine ligands 3 and 4 (CCL3 and CCL4). When TAMs act as M1-like macrophages, they will release pro-IL1, IL6 and IFNγ (**d**). Other subtypes of macrophages can occur depending on the stimuli present in specific tissue or organs (**e**).

Given that TAMs can have multiple origins and functions (see Figure 1), this complicates their ontological classification [10]. Originally, TAMs were described as immune cells that can phagocytose cancer cells and kill them and were therefore defined as anti-tumorigenic [11]. However, it is now widely recognised that TAMs have a dominant tumour-promoting role [12]. The plasticity and diversity of TAMs due to intra-tumoural as well as inter-patient variations complicate identification of consensus markers or ontological classifications that can distinguish tumour-promoting from anti-tumour TAMs [5,10,13,14]. Interestingly, TAMs can be sculpted over time during tumour progression or in response to targeted therapies, or conventional radio-/chemo-therapy [1]. Owing to these biological challenges, the definitions and ontologies of TAMs have been contingent on the dimensionality of analytical techniques. For example, while flow cytometry was initially used to define different TAM subsets, these definitions have since been refined or further complicated by the application of multi-dimensional techniques such as single-cell omics [10,15]. At present, unfortunately, multiple TAMs classification systems co-exist depending on various contexts, e.g., health vs. disease, specific diseases, tissue type or functional activity.

### 2.2. M1- and M2-like Macrophages: A Narrow Vision for a Wide Spectrum of Phenotypes

Macrophages develop into a wide range of phenotypes that can vary from immune defence to immune surveillance. At the polar extremes of this broad range, in vitro stimulated macrophages can acquire a pro-inflammatory phenotype (M1) or an alternatively activated phenotype (M2) (see Figure 1). M1-like macrophages are characterised by their ability to secrete pro-inflammatory cytokines such as interleukin (IL)-1β, tumour necrosis factor (TNF), IL18 and IL12. They express high levels of main histocompatibility complex class II (MHC-II), cluster of differentiation (CD) 68, CD80 and CD86, which are crucial for antigen presentation and co-stimulation [16]. On the contrary, M2-like macrophages play a central role in responses to parasites, tissue remodelling, angiogenesis and allergic diseases. They can be identified by the expression of macrophage galactose-type lectin 1 (MGL1), CD206, CD163, CD200R and CSF1R [16]. 

However, in many instances, there is an overlap in M1 and M2 macrophages and the molecules they express, thereby making it difficult to distinguish them. M1 and M2 denominations refer to two polar opposite polarisation states of macrophages and thus fail to include a vast range of macrophage subpopulations in between [17]. Recent studies using single-cell analysis technologies have even suggested that M1 and M2 signatures can co-exist, highlighting the need for the characterisation of macrophage subpopulation and not just polarisation states [18]. Several macrophage subsets that do not fit M1/M2 definitions have been characterised over the last years, such as CD169^+^ macrophages, STAT1^+^ macrophages, LYVE1^+^ macrophages, SPP1^+^ macrophages, CXCL10^+^ macrophages, FOLR2^+^ macrophages, and regulatory macrophages [16,19,20,21]. Therefore, the division of macrophages according to their inflammatory profiles into pro-inflammatory (M1) and anti-inflammatory (M2) is a simplification, and while widely used and commonly accepted, this division does not fully reflect macrophage or TAMs diversity [9]. 

In addition, most of the conclusions regarding M1/M2 macrophages come from studies in murine models, which often do not fully correspond to observations in humans due to the limited genetic and immunological diversity [4]. Therefore, some of the markers to define M1 and M2 macrophages vary between mouse and human macrophages or are not as specific in humans as in mouse models [22]. This exacerbates the difficulties in translating the findings regarding the prognostic value of macrophages from murine models to human settings. Going forward, a more complex macrophage classification system is required that holistically considers their disease vs. health-specific functions relative to unique cytokine profiles, cytotoxic activity profile (if any) and distinct metabolic states [20]. 

### 2.3. Multi-Faceted Roles of TAMs in Cancer

While TAMs mostly resemble M2-like macrophages, they also share some characteristics of the M1-like phenotype, making this population distinct from a simple M1/M2 division. The role of TAMs in cancer has been controversial. Depending on their phenotype, which is determined by the environment, TAMs can have multi-faceted roles in TME, which, together with their capacity for repolarisation, makes them highly important during anti-cancer therapy [16,23]. TAMs can secrete cytokines and chemokines such as C-C chemokine ligand (CCL) 3, CCL4, TGFβ, and IL10, which recruit regulatory T cells (Tregs) to the TME and suppress the function of CD4^+^ and CD8^+^ T cells, thereby promoting tumour progression [24]. Contrastingly, anti-tumour properties of TAMs have also been identified. In CRC, TAMs showed pro-inflammatory properties and expressed cytokines such as IFNγ, IL6 and IL1, which activated type-1 polarised CD4^+-^ T cells (Th1) cells mediating an anti-tumour immune response [23,25]. Several studies have shown that primarily in CRC, but also to some extent in non-small-cell lung cancer (NSCLC), high TAMs infiltration correlates with increased patient survival [9,23,25]. This might be attributed to the TME in some cancer types that can skew TAMs into M1-like macrophages, e.g., due to gut microbiome in CRC or infiltration of the M1-like macrophages into the tumour islets in NSCLC [18,26]. Conversely, in most cancer types, e.g., breast cancer, ovarian cancer, gastric cancer, renal cell carcinoma and bladder cancer, high TAM infiltration is associated with poor prognosis. Thus it is currently commonly accepted that M2-like TAMs are associated with poor survival in patients and high M1-like TAM infiltration with good prognosis, making repolarisation and reprogramming ability of TAMs a hot topic for research [6,23,27]. 

However, as discussed above (Section 2.2), the heterogeneity of TAMs represents a level of complexity that is not faithfully captured by the simplistic M1- vs. M2-like bifurcation. Indeed, single-cell technologies have helped in distinguishing different phenotypes and context-specific subsets of TAMs and their functional relevance in cancer [1,28]. Such single-cell level distinction of markers differentially expressed by tumour-supporting or immunostimulatory TAMs is important for the development of reliable predictive and/or prognostic biomarkers [1]. For example, Krishna et al. showed that in clear cell renal cell carcinoma (ccRCC) patients, interferon-stimulated genes (ISG)^high^ TAMs could predict better survival after sunitinib treatment [29]. In line with this, House et al. identified a unique macrophage gene signature (including CXCL10 and CXCL11) that was more prevalent in melanoma patients responding to immune checkpoint blockade (ICB) since it was associated with a higher *IFNG* gene signature [30]. These are few illustrative examples of TAM-associated biomarkers on single-cell resolution—a list that keeps expanding as more single-cell mapping initiatives are published. 

As mentioned already, TAMs polarisation reaches a peculiar cross-road with dying cancer cells, especially in the context of anti-cancer therapy. Indeed, M2-like TAMs get attracted to a tumour site with a high burden of dying cancer cells because they have high phagocytosis capacities and will clear the dying cancer cells to facilitate tissue repair [31]. However, the type of cell death induced in a tumour can play an additional role in increasing TAMs heterogeneity and reprogramming. In the following sections, we describe the effect of different types of cell death and cell stress elicited in the TME on TAMs and provide an overview of ongoing research on TAMs as immunotherapy targets. 

## 3. Cell Death in the Context of a Tumour Microenvironment

In a tumour, different cancer cell death modalities occur in a programmed or non-programmed manner, depending on the level and type of stress [32]. Cancer cell death is mainly caused by cellular stress due to DNA damage, endoplasmic reticulum stress, toxins, hypoxia, low levels of glucose and amino acids or other stress inducers triggering programmed cell death (PCD) or non-programmed cell death pathways leading to the release of prominent factors, i.e., cytokines (such as type I IFNs), chemokines (e.g., CCL2, CXCL1) or other danger signals [33,34,35]. 

A major non-PCD pathway is necrosis, which happens in an accidental manner due to stress induced by infection or physico-chemical injury [36]. This type of accidental cell death results in high inflammation caused by the sudden release of intracellular components (including damage-associated molecular patterns, in short DAMPs), which will attract various phagocytes, including TAMs, to clear the dying/dead cells [37]. Herein, the amount and composition of the release DAMPs can be a defining factor behind the M1-like or M2-like activities of TAMs.

On the contrary, PCD is, by definition, a more regulated manner of cell death, based on a mechanistically orchestrated signalling pathway [38]. ‘Physiological’ apoptosis is a commonly known PCD, triggered by different pathways which release ‘find me’ and ‘eat me’ signals, e.g., phosphatidylserine (PS), opsonins, modified intercellular adhesion molecule 3 (ICAM-3) and complement system components recognised by phagocytic cells including TAMs [37,39]. Initially, apoptosis and the cell clearing process were considered as being immunologically ‘silent’ or even tolerogenic, but studies have shown that certain stimuli (which are discussed later in this review) can induce immunogenic apoptosis (also called ‘immunogenic cell death’ or ICD) [40]. Two major routes can induce apoptosis, i.e., the extrinsic (death receptor-elicited) and intrinsic (mitochondrial stress-driven) pathways, both depending on the activity of caspases cleaving and activating downstream cellular substrates [41]. The intrinsic pathway is triggered by a diversity of intracellular stress signals, especially DNA damage or intracellular organellar stress, e.g., cytochrome c, released from the mitochondria [41]. In the cytoplasm, cytochrome c binds and activates apoptosis protease activating factor-1 (Apaf-1) and induces the formation of the apoptosome, which will initiate the cell death pathway via caspases [42]. The extrinsic pathway is activated by extracellular signals engaging their cognate death receptors, e.g., tumour-necrosis factor (TNF) or FAS receptors, which are located on the cellular surface [43]. This interaction leads to the formation of the death-inducing signalling complex (DISC), which activates caspases responsible for the degradation of chromosomes and ultimately leads to apoptosis [43]. 

However, when caspases are inactivated or deficient due to, for example, somatic mutations, a programmed form of necrosis cell death, also called necroptosis, can be activated as a back-up mechanism [38]. This cell death modality has morphological similarities with accidental necrosis but differs in molecular pathways [44]. Since most normal cells engage extrinsic apoptosis, which is less likely to be inflammatory, necroptosis has multiple elements in common with (extrinsic) apoptosis, such as the initiating receptor complexes [45,46]. Whenever extrinsic apoptosis fails to be initiated, proximal initiator receptors of extrinsic cell death (e.g., TNF receptor-1, death receptor 4/5, FAS receptor, Toll-like receptor 3 and 4, Z-DNA binding protein1) will provoke downstream activation of three major pro-necroptotic molecules, i.e., mixed lineage kinase domain such as pseudo kinase (MLKL) and receptor-interacting serine/threonine kinase 1 and 3 (RIPK1/RIPK3) [44]. The activation of these proteins is induced by the engagement of death receptors such as TNF-receptor or TNF-related apoptosis-inducing ligand (TRAIL) and leads to the formation of protein complexes that will eventually cause membrane pores resulting in necroptosis [41]. 

Besides these generally known PCD modes, ferroptosis and pyroptosis are at least two of the many non-apoptotic PCDs that may be found in a tumour [43]. Ferroptosis occurs when the glutathione-dependent antioxidant defence fails via defects in system x_c_^−^ (transports extracellular cystine into cell) or glutathione peroxidase 4 (reduces the cellular level of lipid peroxidation), leading to excessive lipid reactive oxygen species (ROS) that result in necrotic cell death [32]. Lastly, pyroptosis is induced after the activation of inflammasomes through the interaction of particular pattern recognition receptors (PPRs) with DAMPs or other non-infectious agents. This interplay leads to the transcriptional generation of pro-inflammatory mediators such as pro IL-1β, and meanwhile, the inflammasome will be assembled, and caspase-1 activated [47]. The active caspase-1 can proteolytically mature proIL-1β into active forms and hence induce pro-inflammatory-, regulated cell death and pyroptosis [48]. 

## 4. Impact of Tumour Associated Stress and Cell Death on TAMs

**Figure 2:**
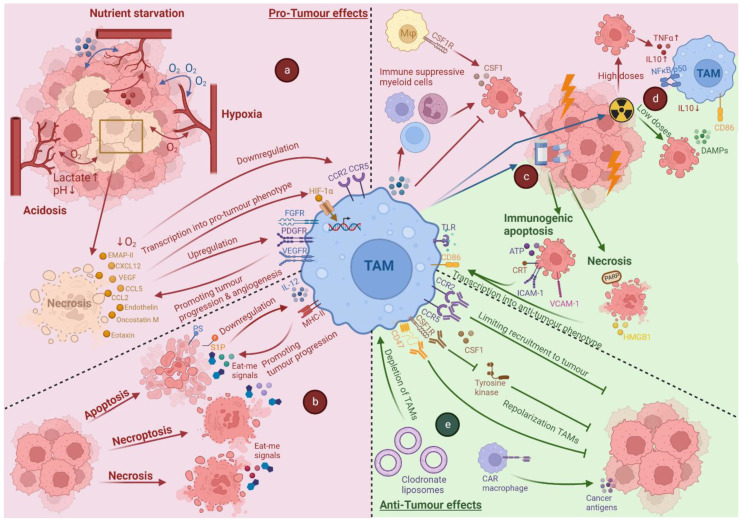
Overview of the impact of cell death due to tumour-associated stress or induced by anti-cancer therapy and the effect of immunotherapy on TAMs. In hypoxic and necrotic areas, recruitment of TAMs occurs through expression of chemoattractant molecules, such as C-C motif chemokine ligands 2 and 5 (CCL2 and CCL5), C-X-C motif chemokine ligand 12 (CXCL12), endothelin, vascular endothelial growth factor (VEGF), endothelial monocyte-activating polypeptide-II (EMAP-II), oncostatin M and eotaxin. Once in hypoxic areas, the conditions will downregulate C-C motif chemokine receptors 2 and 5 (CCR2 and CCR5) causing retention and entrapment of TAMs in the tumour. This will induce transcription in TAMs by upregulating hypoxia-inducible factors-1 and -2 (HIF-1, HIF-2). Simultaneously, hypoxia can promote surface expression of VEGF receptor, platelet-derived growth factor receptor (PDGFR) and fibroblast growth factor receptor (FGFR). Besides hypoxia, acidosis and nutrient starvation can both affect macrophage metabolism leading to pro-tumour effects (**a**). Cancer cells undergoing apoptosis will expose eat-me signals, such as phosphatidylserine (PS) and sphingosine-1-phosphate (S1P), which can reduce production of interleukin 12 (IL 12) and downregulate main histocompatibility complex-II (MHC-II) presented by TAMs. Both accidental and programmed necrosis (i.e.,) necroptosis, secrete eat-me signals that will cause a pro-tumour effect (**b**). Chemotherapy can induce cell death, resulting in high expression of colony-stimulating factor 1 (CSF1) on the cancer cells. This will attract CSF1-receptor (CSF1R) positive macrophages together with TAMs, which secrete stimulatory factors to recruit other immune-suppressive myeloid cells. However, certain chemotherapies can induce immunogenic apoptosis leading to exposure/secretion of calreticulin (CRT), intercellular adhesion molecule (ICAM)-1 and vascular cell adhesion molecule (VCAM)-1 and adenosine triphosphate (ATP). When deficiency in the apoptosis pathway, necrosis is stimulated, and the interaction of released high mobility group box 1 (HMGB1) with Toll-like receptors (TLRs) triggers pro-inflammation (**c**). Depending on the doses, radiotherapy can reprogram TAMs to a phenotype sustained by nuclear factor kappa-light-chain-enhancer of activated B cells B (NFκB) p50 activation that leads to IL10 and tumour necrosis factor α (TNFα) secretion. Low doses can induce secretion of damage-associated molecular patterns (DAMPs) and upregulation of cluster of differentiation 86 (CD86) (**d**). Limiting of TAMs at tumour sites can be performed by blocking C-C chemokine receptors 2 and 5 (CCR2 and CCR5). Repolarisation of the TAMs is performed by blocking the CSF1-CSF1R interaction through antibodies, by inhibiting tyrosine kinases or by blocking CD47. Depleting TAMs can be performed by using clodronate liposomes to induce apoptosis. Lastly, CAR macrophages can induce phagocytosis of cancer antigens (**e**).

As discussed previously, cell death occurs continuously in the tumour, mostly as a result of cell stress. The stressful conditions in the TME (e.g., metabolic stress, hypoxia) and the cross-talk with dying cancer cells in the tumour have deep effects on TAM phenotype and polarisation and play a major role in tumour progression (see Figure 2).

### 4.1. Impact of Hypoxia on TAMs

Hypoxia is a major cause of cell stress and death in the tumour, arising early in tumourigenesis and is a major driver of TAM recruitment and polarisation [49,50,51,52]. Moreover, hypoxia also generates pro-tumorigenic signals and is a key factor in tumour growth and metastasis [53,54]. 

The accumulation of TAMs at high densities in hypoxic and necrotic areas of tumours has been observed in multiple cancer types [55,56]. Hypoxic cells upregulate the expression of chemoattractant molecules, such as CCL2, CCL5, CXCL12, endothelin, vascular endothelial growth factor (VEGF), endothelial monocyte-activating polypeptide-II (EMAP-II), oncostatin M and eotaxin, attracting monocytes and macrophages to these areas [49,52,57,58,59,60,61,62,63]. Once in these areas, the hypoxic conditions cause downregulation of the receptors for several of these factors, such as CCR2 and CCR5, contributing to the retention and entrapment of TAMs in the tumour [64].

TAMs are affected by the lack of oxygen and by the signals released by hypoxic tumour cells, undergoing transcriptional changes resulting in a pro-tumour phenotype. In part, this is due to hypoxia-induced upregulation of transcription factors that are sensitive to oxygen, hypoxia-inducible factors-1 and -2 (HIF-1 and HIF-2), which regulate transcription of several oxygen-sensitive genes [65,66]. HIF-regulated genes help cells survive in low oxygen conditions by promoting angiogenesis, increasing glycolysis, and suppressing oxygen consumption. 

Currently, although the correlation between HIF signalling and the TAM M2-like phenotype is very strong, most data suggest that HIF may not always directly drive the differentiation of TAMs into an M2-like phenotype [67]. Instead, hypoxia might in some cases fine-tune the pre-existing TAM phenotype by upregulating growth factors that promote tumour growth and tumour angiogenesis, such as (VEGF), platelet-derived growth factor (PDGF) and fibroblast growth factor (FGF) [67,68]. Additionally, TAMs, and in particular hypoxic TAMs, promote tumour invasion and tumour cell migration by releasing enzymes that degrade the extracellular matrix, such as matrix metalloproteinases (MMPs) [69,70]. 

The importance of tumour hypoxia in regulating the immunosuppressive activity of TAMs has been shown by the specific deletion of the receptor neuropilin1 (Nrp1) in macrophages [71]. This study showed that Semaphorin3a, which is induced by hypoxia, attracts TAMs to the hypoxic regions of the tumour through Nrp1, triggering PlexinA1/A4-dependent VEGF receptor 1 (VEGFR1) activation. Genetically targeting Nrp1 impedes the entry of TAMs into hypoxic regions, which are retained in the normoxic regions instead, preventing immunosuppression and angiogenesis and promoting anti-tumour immunity. This shows not only the importance of hypoxic TAMs but also that TAM heterogeneity is directly related to their spatial localisation in the tumour [71]. 

### 4.2. The Impact of Acidosis and Nutrient Starvation on TAMs

Along with, and sometimes due to, hypoxia, the metabolism of tumour cells also contributes to the immunosuppressive TME through acidosis. Tumour cells, unlike normal cells, predominantly produce energy and use glucose via aerobic glycolysis, a phenomenon known as the “Warburg effect”. This results in the consumption of oxygen and excessive production of hydrogen ions (H^+^) and lactic acid, leading to a hypoxic and acidic TME.

Lactic acid promotes TAMs’ pro-tumour functions in multiple ways. Lactic acid is sensed by the G-protein coupled receptor 132 (GPR132), which, upon activation, promotes an M2-like phenotype [72]. In turn, lactate activates GPR81, which is present in a wide array of cell types and has been shown to be highly expressed in various cancer cell lines, where its activation promotes cell survival and tumour progression via different mechanisms. These include the upregulation of monocarboxylate transporters, amphiregulin, ATP Binding Cassette Subfamily B Member 1 (ABCB1) and programmed-death ligand 1 (PD-L1) [73,74,75]. Finally, lactic acid in the TME also activates HIF-1α and -2α in TAMs, leading to the aforementioned hypoxia-related pro-tumour effects [76,77]. A recent finding from us showed that lactic acid regulates TAM polarisation by pH modification, but it does so only in conjunction with a specific cytokine milieu [78]

The limited nutrient availability inside the TME also affects the metabolism of TAMs. TAMs are dependent on the glutamine-glutamate pathway for their energy requirements and show increased expression of glutamate transporter genes and glutamine synthase [79]. The release of glutamine by TAMs that can then be used by cancer cells has also been described in glioblastoma [79,80]. Targeting this pathway can shift TAM polarisation towards an M1-like phenotype [81,82]. At low arginine concentrations, ARG1 and inducible nitric oxide synthase (iNOS) are expressed in TAMs, favouring the production of reactive oxygen/nitrogen species, which further inhibit immune function in the TME [83]. However, lactate also has been shown to differentially affect TAM subsets and TAM transcriptome in a GPR132-independent manner, increasing the immunosuppressive capacity of MHC-II^low^ TAMs [13]. 

Furthermore, TAMs are described to strongly express indoleamine-pyrrole 2,3-dioxygenase (IDO1), which promotes immunosuppression directly by tryptophan depletion and indirectly through the production of metabolites such as kynurenine [84,85,86]. Finally, the accumulation of lipids in TAMs, which is also partly caused by hypoxia, is known to regulate their function and promote the immunosuppression of T cells [87,88,89]. Recently, receptor MARCO has emerged as an important mediator of this process in TAMs and targeting it has been shown to reduce tumour growth [90]. 

In summary, the hypoxic and acidic TME affects macrophage metabolism and contributes to the immunosuppressive functions of TAMs.

### 4.3. Cross-Talk between Dying Cancer Cells and TAMs

Death of cancer cells as a result of metabolic stress directly influences TAMs metabolism and phenotype. The different cancer cell death pathways can result in either a pro- or anti-tumour response in immune cells.

The role of apoptosis as an immunosuppressive, pro-tumorigenic mechanism is well established. Indeed, apoptotic cancer cells manipulate TAM function to support tumour growth and progression [91,92]. Apoptotic cells are drivers of M2-like polarisation and suppress the inflammatory activity of M1-like TAMs, effectively suppressing anti-tumour immunity [93,94]. During apoptosis, membrane structure is altered, and eat-me signals are exposed at the surface. Annexin I and PTX3, among others, interact directly or indirectly with phagocytes [95,96]. The most significant change in the lipid composition of the membrane is the oxidation and transfer of phosphatidylserine (PS) from the inner to the outer leaflet [97,98]. Recognition of surface PS by TAMs is associated with altered macrophage function and tumour progression [99]. Not only surface eat-me signals but also soluble factors produced by apoptotic cells impact the TAM response in a seemingly overlapping way. Sphingosine-1-phosphate (S1P), for instance, is released by apoptotic cells and acts to attract and activate TAMs. S1P has been shown to induce a pro-tumour phenotype in TAMs by reducing the production of cytokines such as IL12 and downregulating MHC-II expression [93,100]. Additionally, S1P can also activate HIF-1a, resulting in the production of VEGF and pro-tumour cytokines such as IL10 and IL6 [100,101,102]. Finally, S1P reduces CD80 expression on TAMs by inducing the production of prostaglandin E2 [103,104]. 

Necrosis, both accidental and programmed (i.e., necroptosis), can happen in the tumour due to the variety of stressors mentioned above, i.e., hypoxia and acidosis [44]. These modalities of cell death are considered to be typically inflammatory, resulting in the release of DAMPs. Necroptosis has a controversial role in cancer, seemingly dependent on the tumour type, which is further complicated by the difficulties in correctly identifying necroptosis in vivo [105]. Necrotic and necroptotic cancer cells can promote tumour development by recruiting TAMs to the TME and releasing regulatory cytokines, such as IL1α, that promote cancer cell proliferation [106]. 

In summary, the extreme metabolic conditions and resulting continuous cell death of cancer cells happening in the tumour microenvironment promote pro-tumour responses in TAMs and play a major role in tumour progression. 

## 5. Anti-Cancer Therapy-Induced Cell Death and Its Impact on TAMs 

Chemotherapy, radiotherapy or targeted therapies are the most prominent cell-death-inducing conventional therapies, and they exert dual effects depending on the type of therapy and their dosage schedules. One of the most important effects is ICD, which causes the release of pro-inflammatory cytokines, tumour specific/associated antigens, and DAMPs and thereby triggers immune cells to elicit anti-tumour immune responses [107,108]. Most of these therapies are designed to interfere with pathways necessary for tumour growth and progression [109]. For chemotherapy, there are different classes with either drastically or subtly different mechanisms of action, primarily entailing direct or indirect DNA damage-based apoptosis [110]. The ability of these chemotherapeutic agents, e.g., anthracyclines, oxaliplatin, and paclitaxel, among others, to convert an immune-silent or immuno-suppressive TME into a more immunogenic TME by releasing DAMPs is a very hot topic in immuno-oncology [111]. Furthermore, radiotherapy can be used to induce ICD via apoptosis or necroptosis, leading to a shift in the TME from immunosuppressive towards one that incites an anti-tumour immune response as well as abscopal effect (i.e., the ability to regress distant, non-irradiated, tumour lesions) [112,113]. During cancer therapy, TAMs can contribute to the efficacy of anti-cancer strategies or promote tumour formation [25]. Depending on the therapy given and, therefore, the type of cell death induced, TAMs will react differently. Below, we discuss the impact of chemotherapy- and radiotherapy-induced cell death on TAMs.

### 5.1. Impact of Chemotherapy-Induced Cell Death on TAMs

Chemotherapy, apart from inducing cancer cell death, also causes general tissue damage, all of which together results in the recruitment of TAMs to ‘repair the tissue’. 

Chemotherapy-induced cancer cell death can have a repolarisation effect on TAMs, depending on their mechanism of action. Taxanes (like paclitaxel and docetaxel) induce cell cycle arrest, which eventually leads to cancer cell death. This stimulates TAMs to induce pro-inflammatory genes and repolarise them into M1-like TAMs with anti-tumoural effects [23]. Alkylating agents (like cisplatin and carboplatin) inhibit DNA replication from causing cancer cell death, wherein such dying cancer cells express more CSF1, thereby attracting large numbers of CSF1R^+^ TAMs (M2-like) [114]. The TAMs in the TME also enhance the production of IL1β and TNF and activate the NF-κB pathway, skewing the differentiation of TAMs towards an M2-like phenotype [23,115,116]. Anthracyclines (like doxorubicin) inhibit RNA and DNA synthesis and cause ICD in cancer cells, leading to the repolarisation of TAMs towards an M1-like phenotype. Lastly, antimetabolites (like 5-fluorouracil and gemcitabine) also disrupt RNA and DNA synthesis and thereby facilitating the M1 phenotype in TAMs [23]. 

Conventionally chemotherapy was given at a maximum tolerated dose (MTD), but to overcome adverse effects, lower doses or metronomic chemotherapy (MCT) are being increasingly used [117]. Considerable evidence exists that MCT can induce ICD [117]. ICD results in calreticulin (CRT) exposure on the surface of dying cells, which is recognised as an eat-me signal by phagocytes to clear these cells. CRT also increases expression of intercellular adhesion molecule (ICAM)-1 and vascular cell adhesion molecule (VCAM)-1 on tumour endothelial cells via nuclear factor kappa-light-chain-enhancer of activated B cells (NF-κB) activity, thereby improving anti-tumour immune responses [118,119]. ATP is another molecule released by cancer cells after ICD, which functions as a find-me signal, but also binds to ionotropic P2X purino-receptor 7 (P2RX7), inducing inflammasome activation and IL1β release [120]. This leads to a more effective anti-tumour immune response and tumour immunosurveillance [92]. In a study looking at the effect of MCT and MTD on the polarisation of TAMs, it was found that the MCT group facilitated M1-like TAMs and inhibited M2-like TAMs, compared to the MTD group [117]. 

In summary, the recruitment and repolarisation of TAMs towards an M2-like phenotype will promote the recruitment of immune-suppressive myeloid cells, such as CSF1R^+^TAMs and myeloid-derived suppressor cells (MDSC), ultimately suppressing the adaptive anti-tumour immune responses and thereby hampering the chemotherapy efficacy [121]. By secreting growth factors and inhibiting cell death signalling pathways in tumour cells, TAMs can also actively contribute to chemoresistance. In a way, chemotherapy can be said to be counterproductive since it causes the attraction of M2-like macrophages, which will cause tumour progression [25]. Yet, if the given chemotherapy is inducing ICD inducing, it will change the phenotype of TAMs to more inflammatory M1-like TAMs, thereby suppressing tumour growth [23]. As such, depending on the class and dose of chemotherapy given, the TME will change depending on the type of induced cell death. TAMs can therefore either contribute to anti-tumour activity or promote tumour progression. 

### 5.2. Impact of Radiotherapy-Induced Cell Death on TAMs

The effect of radiotherapy-induced cancer cell death on TAMs depends on the dose, frequency, and localisation of the radiotherapy. These are crucial to either induce stimulation or inhibition of the immune responses [122]. 

Dying cancer cells generated by a single high dose of radiation therapy can cause M2-like polarisation of newly recruited macrophages or reprogramming towards an M2-like phenotype. This phenotype is sustained by NF-κB p50 activation, leading to IL10 and TNF secretion [123]. Yet, dying cancer cells through single moderate doses of irradiation have the opposite effect on TAMs. They shift them towards an M1 phenotype, highlighted by the upregulation of pro-inflammatory markers (like CD86), downregulation of anti-inflammatory markers (like CD163, CD206) and reduction in IL10 secretion [122]. This can be explained by the release of DAMPs from cancer cells after moderate doses of irradiation, which ultimately triggers an anti-tumour response as described above [121,122]. To avoid toxicity observed with a single high dose of irradiation, fractionated low doses are also given. However, cancer cell death induced by fractionated low doses of radiation has been shown to decrease iNOS level and NO production, leading to a decrease in IL1β, which favours M2-like macrophages [124]. 

The effects of irradiation on TAMs reprogramming need attention due to its contradictory impact. It is important to consider if an anti-inflammatory or pro-inflammatory TME is created after radiotherapy. Therefore, it might also be wise to choose a combination of lower doses of radiotherapy with ICD-inducing chemotherapy to create an inflammatory immune reaction actively killing the tumour cells (currently more clinically plausible) or to combine either chemotherapy or radiotherapy with immunotherapy targeting TAMs, to increase the anti-tumour reaction (a pre-clinical paradigm that requires clinical validation) [122].

## 6. Immunotherapy Targeting TAMs 

As described above, TAMs are greatly influenced by the dying cancer cells, which is intensified by anti-cancer therapies, and in turn, can also contribute to immunotherapy resistance (Expanded in Box 1) [125].

Box 1 **The role of tumour-associated macrophages (TAMs) in response to immune checkpoint therapy.**

Immune checkpoint blockers (ICBs) such as anti-PD1, anti-PDL1 or anti-CTLA4 ICBs have mobilized a major paradigm-shift in the treatment of solid tumours [113]. Although ICBs do not directly induce cancer cell death like conventional anticancer therapies, yet they indirectly cause cancer cell death through activation of cytotoxic CD8^+^ T cells. Many reviews have described the role of TAMs in ICB resistance [1].

TAMs express various immune checkpoint ligands or receptors, such as PDL1, PDL2, CD80, CD86, PD1 and TIM3, through which they elicit immune suppression in cancer [113]. Upregulation of these immune checkpoint ligands and receptors is associated with an immunosuppressive M2-like phenotype, thereby contributing to tumour promotion and therapeutic resistance [113]. Reducing or blocking the expression of these ligands/receptors on TAMs (e.g., via ICBs) can change them to a more M1-like phenotype with immune-stimulation and cytotoxic activities [113,126,127].

Several studies have shown a specific role for TAMs in resistance to ICBs. Koh et al. showed that in RCC, anti-inflammatory M2-like TAMs were associated with resistance to ICB [128]. In human and murine non-small-cell lung cancer (NSCLC) tumours, Peranzoni et al. showed that, CD8^+^ T cells migrate poorly to the tumour site due to interaction with TAMs in the stroma. Depletion of TAMs was able to restore CD8+T cell migration and infiltration to the tumour, thereby improving anti-PD1 ICB efficacy [129]. Another study showed that in NSCLC patients specifically TIM4^+^ TAMs suppress CD8^+^ T cells and inhibit ICB efficiency, which can be reversed by blocking TIM4 [130].

Similar to conventional anti-cancer therapies, M1-like TAMs play a role in better response to ICB. For example, Zeng et al. showed that anti-PDL1 ICB response could be predicted in urothelial cancer when there was high infiltration of M1-like TAMs [131]. In line with this study, House et al. identified a unique TAMs gene signature (including CXCL10 and CXCL11) that was more present in melanoma patients responding to ICB, since it was associated with a higher IFNγ gene signature [30].

However, single markers can often be of low predictive value and might still not explain why ICB works successfully in one patient and not the other. Since response to ICB is dictated by a very complex network of cellular interactions between cancer and immune cells, Antoranz et al. showed that spatial distribution of cell types should also be considered. They recently showed that spatial distribution of PDL1^+^ TAMs and cytotoxic T cells could predict response of melanoma patients to anti-PD1 ICB [132].

For this reason, combining traditional therapies together with TAM-targeting therapies has been emphasised over the last decade [133]. So far, there are three main strategies for targeting the TAMs to overcome their pro-tumour properties: (I) Limiting their recruitment; (II) repolarisation toward M1-like TAMs; and (III) total TAM depletion (see Figure 2). Considering a large amount of TAM targeting strategies, we focus our further discussion on only those approaches that have been, or are currently being, tested in a human context (for some major ongoing clinical trials in this respect, please see Table 1). Of note, it is important to keep in mind that these therapies are always used together with cell death inducers. Therefore, the results from these agents are always intertwined.

### 6.1. Limiting the Recruitment of TAMs 

In order to reduce TAM accumulation at the tumour site, antagonists against chemokine receptors, such as CCR2, have been developed. CCR2 functions as a receptor for CCL2 and is mainly expressed by TAMs, monocytic cells, dendritic cells (DCs) and endothelial cells but also neutrophils and lymphocytes [134]. CCL2 binding to its receptor has an anti-apoptotic effect and promotes angiogenesis and cell migration [135,136]. It has been suggested that blocking the CCL2-CCR2 axis can inhibit the recruitment of TAMs to the tumour site. Initial studies inhibiting CCR2 by the use of antibodies have shown reduced tumour growth and improved efficacy of chemotherapies in multiple murine tumour models [137]. Unfortunately, clinical trials using CCR2 targeting agents, such as carlumab, a human anti-CCR2 antibody, alone or in combination with chemotherapy, resulted in only a short-term CCR2 suppression in the serum and no anti-tumoural response [138]. Additionally, it has been shown that the destabilisation of CCR2 after therapy can cause increased cancer progression [139]. Additionally, CCR2/CCR5 dual inhibitors have been put forward as a potential manner of decreasing TAM recruitment. Presently, a phase I/II clinical trial is testing the potential of BMS-813160, anti-CCR2/CCR5, in colorectal and pancreatic cancer (NCT03184870).

Another pathway targeted to limit TAM accumulation is the CXCL12/CXCR4 axis. The binding of CXCL12 to CXCR4 is involved in multiple biological processes such as proliferation, angiogenesis and metastasis of cancer [140]. Inhibiting CXCR4 with AMD3100/plerixafor has been tested in clinical trials for patients with colorectal or pancreatic cancer. Indeed, AMD3100/plerixafor did increase the amount of intra-tumoural T cells, although the limited treatment period in these particular studies did not result in any treatment response [141]. Furthermore, multiple similar studies using different CXCR4 antagonists have been started and are currently ongoing [142].

### 6.2. Repolarisation of TAMs

The second mode of action for TAM-targeted immunotherapy is reprogramming the TAM compartment to be more immunogenic and anti-tumoural. Presently, a lot of efforts have been directed at inhibiting the CSF1R-CSF1 axis. This is either achieved by the use of monoclonal antibodies against CSF1R or CSF1 or by the inhibition of tyrosine kinases downstream of CSF1R. Current CSF1R targeting therapies have been reviewed in depth elsewhere [143,144]. As of yet, there has not been a consensus about the mode of action of CSF1R inhibition. Some studies have shown evidence that suppressing the CSF1R-CSF1 axis will repolarise the TAM compartment [145] while others demonstrate partial depletion of the M2 TAMs [114]. Many clinical trials are targeting the CSF1R axis as monotherapy but also in combination with treatment regimens such as chemotherapy are currently ongoing. So far, there have only been some anecdotal reports of clinical responses. For example, 3 out of 146 patients with solid tumours receiving BLZ945 showed partial response [146]. Furthermore, additional analysis of peripheral blood monocytes did not show any changes after treatment [147,148].

Another strategy to block the immune suppressive properties of M2 TAMs is by controlling the TAM polarisation. Toll-like receptor activation is considered to be an attractive strategy for achieving more M1-like polarisation [149,150]. Multiple TLR agonists are currently being used in clinical trials as an anti-cancer treatment, although they are not specifically targeting TAMs [151,152,153]. Currently, efforts have been made to develop strategies more targeted towards TAMs, such as R848, TLR agonists, or nanoparticles containing mRNA or TLR agonists showing promising results [154,155,156,157].

Additionally, increasing phagocytosis by blocking CD47, a well-known ‘do notx eat me’ signal (i.e., efferocytosis inhibitor), has been shown to cause TAM repolarisation [158]. This surface ligand found on all cells modulates efferocytosis by interacting with the signal regulatory protein alpha (SIRPα) on TAMs [159]. When a target for phagocytosis expresses CD47, the internalisation step of phagocytosis gets inhibited, blocking phagocytosis altogether [160]. Using CD47 targeting antibodies such as magrolimab, one can increase phagocytosis and therefore enhance the M1 phenotype. However, the use of anti-CD47 was not effective as a monotherapy. However, the combination of magrolimab with an anti-CD20 antibody caused a 36% complete response rate in non-Hodgkin’s lymphoma (NHL), albeit in a small cohort [161]. So far, multiple phase I clinical trials focusing on CD47 suppression have been initiated and have shown limited results, largely due to toxicity concerns due to CD47’s ubiquitous expression in many non-tumour organ systems [162,163,164,165]. Finally, anti-SIRPα clinical trials have also started to address the concerns about the side effects of anti-CD47 immunotherapy [166].

### 6.3. Depletion of TAMs 

As mentioned above, CSF1R targeting studies have reported a partial depletion effect in the TAM compartment. For this reason, a large portion of the TAM-depleting studies are using CSF1R targeting therapies. Higher concentrations of CSF1R blockers have been found to be depleting the TAMs as well as the resident macrophages [167]. In general, more off-target effects can be expected from high-dose therapies, something that is not yet clear for anti-CSF1R. Apart from anti-CSF1R, one of the chemicals for TAM depletion used in pre-clinical models is clodronate liposomes. Liposomes encapsulating clodronate are endocytosed by phagocytes. Since the clodronate cannot cross the phospholipid bilayer, it will accumulate, ultimately causing apoptosis. However, this approach is non-specific and highly cytotoxic because of its capacity to target not only pro-tumoural macrophages but also the entire phagocyte compartment. 

### 6.4. CAR-Macrophages

The use of chimeric antigen receptor (CAR) macrophages as an anti-cancer therapy is one of the most recent approaches being explored. CARs are synthetic receptors bioengineered to recognise specific target antigen. Generally, T cells are used as a host for the CAR to generate a robust T-cell response. Similar to T-cell CARs, Macrophage CARs consist of an extracellular domain for specific antigen recognition, a hinge domain, a transmembrane domain of CD8α and an intracellular domain of FCER1G, MEGF10, MERTK or CD3ζ molecules for downstream signaling [168]. Binding to the CAR induces phagocytosis of the cancer cells with the specific antigen, which is subsequently presented to T cells to initiate anti-cancer immunity [169]. Momentarily, the second generation of CAR macrophages is being developed that focuses on the improvement of T-cell activation and antigen presentation. Additionally, special attention is being paid to potentiating the anti-cancer phenotype of the CAR macrophages [168,170]. Presently, a first in human clinical trial is investigating the therapeutic potential of CT-0508, autologous second-generation macrophages expressing an anti-HER2 CAR, in metastatic solid cancers (NCT04660929). So far, mouse studies have revealed that CT-0508 causes phagocytosis of cancer cells specifically, thereby decreasing tumour growth and increasing survival [171]. However, its efficiency in the human context remains to be determined.

Of note, although the United States (US) Food and Drug Administration (FDA) has granted a fast development track for the CT-0508 modality, there are still some concerns. At this point, upscaling CAR macrophages is difficult since there is still an expansion problem. For this reason, novel strategies are being created that utilise pluripotent stem cells (iPSCs) that can be differentiated towards myeloid/macrophage lineages [172]. We believe if these obstacles are overcome soon, the next generation of CAR-macrophages can become a very promising anti-cancer therapy.

## 7. Conclusions

TAMs engage in a complex interface with dying cancer cells. Depending on the spatial (relative to hypoxic, necrotic, core or invasive regions), temporal (duration of therapy or tumour-associated stress), quantitative (amount of cell death or stress, dosage of anti-cancer therapy) and qualitative (type of cell death, type of anti-cancer therapy or cancer-type) aspects of this interface, TAMs may exert pro-tumorigenic or anti-tumorigenic activities. Thus, TAMs play a multi-faceted role in modulating tumour growth and the efficacy of chemo-radiotherapy or immunotherapy. Despite their crucial tumour and therapy modulatory impact, therapeutic modulation of TAMs has not yielded highly successful clinical results as yet. Herein, the extreme TAMs heterogeneity, plasticity, human vs. mouse variations and context-dependent activity together serve as major bottlenecks for the therapeutic translation of TAM-targeting immunotherapies. Multi-omics dissections of the human TAM compartment and analyses of conserved mechanisms and ontologies between human and mouse TAMs are together expected to provide crucial insights into the next generations of TAM-targeting immunotherapies [173,174]. Nevertheless, it is necessary for future TAMs-targeting strategies to sufficiently account for the tissue contexture of tumours as well as metastasis. Herein, modulation of the TAM-dying cancer cell interface by targeting phagocytic pathways (e.g., CD47-SIRP1α, CAR-macrophages) is poised to be the next big breakthrough in immuno-oncology, contingent on successful results in ongoing clinical trials.

## Figures and Tables

**Table 1:** Selected ongoing clinical trials evaluating major macrophage-targeting therapies.

Mechanism/Target	Cancer Type	Combination Agents	Identifier
CCR2/CCR5	Pancreatic adenocarcinoma	Radiation, nivolumab, GVAX	NCT03767582
	NSCLC, hepatocellular carcinoma	Nivolumab	NCT04123379
	Colorectal cancer, pancreatic cancer	Nivolumab, ipilimumab	NCT04721301
	Colorectal cancer, pancreatic cancer	Nivolumab, chemotherapy	NCT03184870
CXCL12/CXCR4	Multiple myeloma	G-CSF	NCT03246529
	Pancreatic adenocarcinoma	Cemiplimab	NCT04177810
	Pancreatic adenocarcinoma	Pembrolizumab	NCT02907099
	Pancreatic adenocarcinoma	Cemiplimab, gemcitabine, nab paclitaxel	NCT04543071
CSF1/CSF1R	Solid tumours	PDR001	NCT02829723
	Sarcoma	Sirolimus	NCT02584647
	Sarcoma, neurofibroma, leukaemia	-	NCT02390752
	Melanoma	Vemurafenib, cobimetinib	NCT03101254
	Breast cancer	Nivolumab, paclitaxel, carboplatin	NCT04331067
	Leukaemia	-	NCT03922100
CD47/SIRPα	Myelodysplastic syndrome	Azacitidine	NCT04485065
	Hodgkin Lymphoma	Pembrolizumab	NCT04788043
	B-cell lymphoma	Venetoclax, obinutuzumab	NCT04599634
	Solid tumours	-	NCT04900519
	Myelodysplastic Syndrome	Azacitidine	NCT04900350
	Solid tumours	BI 754091	NCT03990233
	Solid tumours, lymphoma	-	NCT04306224
CAR macrophages	Solid tumours	-	NCT04660929

CAR, chimeric antigen receptor; CSF1, colony-stimulating factor 1; CSF1R, colony-stimulating factor 1 receptor; G-CSF, granulocyte colony-stimulating factor; NSCLC, non-small-cell lung cancer; SIRPα, signal regulatory protein alpha.

## Data Availability

Not applicable.
